# Correlating charge and thermoelectric transport to paracrystallinity in conducting polymers

**DOI:** 10.1038/s41467-020-15399-2

**Published:** 2020-04-08

**Authors:** Anas Abutaha, Pawan Kumar, Erol Yildirim, Wen Shi, Shuo-Wang Yang, Gang Wu, Kedar Hippalgaonkar

**Affiliations:** 1 Institute of Materials Research and Engineering, Agency for Science Technology and Research, #08-03, 2 Fusionopolis Way, Innovis, Singapore, 138634 Singapore; 2 Institute of High Performance Computing, Agency for Science, Technology and Research, 1 Fusionopolis Way, #16-16 Connexis, Singapore, 138632 Singapore; 3 Department of Chemistry, Middle East Technical University, 06800 Ankara, Turkey; 4 Materials Science and Engineering, Nanyang Technological University, 50 Nanyang Avenue, Singapore, 639798 Singapore

**Keywords:** Thermoelectric devices and materials, Electronic devices, Polymers, Electronic properties and materials

## Abstract

The conceptual understanding of charge transport in conducting polymers is still ambiguous due to a wide range of paracrystallinity (disorder). Here, we advance this understanding by presenting the relationship between transport, electronic density of states and scattering parameter in conducting polymers. We show that the tail of the density of states possesses a Gaussian form confirmed by two-dimensional tight-binding model supported by Density Functional Theory and Molecular Dynamics simulations. Furthermore, by using the Boltzmann Transport Equation, we find that transport can be understood by the scattering parameter and the effective density of states. Our model aligns well with the experimental transport properties of a variety of conducting polymers; the scattering parameter affects electrical conductivity, carrier mobility, and Seebeck coefficient, while the effective density of states only affects the electrical conductivity. We hope our results advance the fundamental understanding of charge transport in conducting polymers to further enhance their performance in electronic applications.

## Abstract (web summary)

Obtaining a complete picture for charge transport in conducting polymers is vital to designing new organic electronic materials. Here, the authors show that a gaussian density of states clarifies the transport physics in conducting polymers by revealing the role of carrier scattering on transport.

## Introduction

The diverse morphologies obtained from different processing methods obfuscate the fundamental understanding of charge transport in conducting polymers. These morphologies alter the degree of energetic disorder in the electronic structure significantly^1,2^. The structural disorder is usually described by paracrystallinity (*g*) which represents the fluctuation range of interchain spacings. A general relationship between charge transport and paracrystallinity in conducting polymers is known^1^, showing that higher *g* induces more states in a material’s electronic band gap, which limits charge transport in conducting polymers^2^. Those electronic states were shown to distribute in a Gaussian shape in energy space^3,4^, where its width (*w*) is defined as energetic disorder. Although there have been some efforts to establish a charge transport model based on Gaussian DOS^5^, energy-dependent scattering of the charge carriers was ignored, which is crucial in determining transport, especially in highly doped polymers that are useful for real-world applications. Experimentally, thermoelectric studies provide a rigorous approach to probe energetics of charge scattering^6^ with respect to structural morphology^7,8^. For instance, energy-dependent scattering was considered in describing charge transport in conducting polymers; however, the DOS was not accounted for, resulting in partial understanding of charge transport^6^.

Energetic disorder in conducting polymers is caused by many factors such as positional disorder^1^, dynamic effects^9^, polarization, and polaronic effects^10,11^. However, their contributions to the total energetic disorder vary depending on the organic system under study. For example, polarization effect, due to induced dipole moments, is a major cause of the energetic disorder in small molecules where positional disorder is almost neglected^11^. On the other hand, polaronic effect due to doping in organic semiconductors could alter the energetic disorder; however, it can be negligible in intrinsically highly disordered polymers^10^. Therefore, positional disorder plays the most important role in conjugated polymers with higher DOS widths (*w* > 0.1 eV)^10^.

In our work, we account for such structural disorder by using Gaussian DOS in the Boltzmann Transport Equation (BTE) under relaxation time approximation. Several scattering mechanisms play a key role in shaping charge transport as defined by BTE formalism. The scattering parameter (*r*) is a physical factor that reflects a specific scattering mechanism. We find that *r* affects the electrical conductivity, carrier mobility, and Seebeck coefficient, while the effective DOS only affects the electrical conductivity. We investigate the relationship between transport properties and the DOS determined by paracrystallinity in conducting polymers. First, we perform tight-binding (TB) model calculations supported by density functional theory (DFT) and molecular dynamics (MD), to confirm that the DOS tail exhibits a Gaussian shape whose width (*w*) increases exponentially with *g*. Second, we corroborate our model by fitting literature data of transport properties for a variety of conjugated polymers, and conclude that different classes of these possess distinct *r* values and effective DOS.

## Results

### Electronic density of states and paracrystallinity

**Fig. 1:**
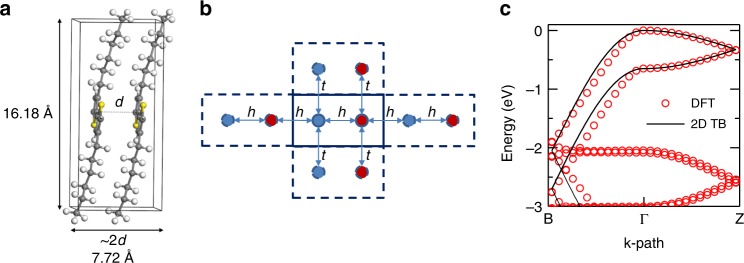
Electronic band structure of poly(3-hexylthiophene) (P3HT) obtained by density functional theory (DFT) and tight-binding (TB) calculations. **a** Lattice structure of P3HT unit cell consisting of two chains with two monomers each. **b** A two-dimensional (2D) TB model where the red and blue circles stand for the two inequivalent sites in each P3HT unit cell. **c** Comparison of the electronic band structures of P3HT obtained from DFT and 2D TB model.

Charge transport along polymer backbones (intrachain) is favorable due to stronger electronic coupling within the chain; however, the electronic coupling between the backbones (interchain, π–π) is more critical since it dominates macroscopic transport properties in a real polymeric system^12,13^. So, the effect of *g*, in the interchain direction, on *w* is more relevant to our study. As an example, we explore the electronic band strucure of the prototypical conducting polymer, poly(3-hexylthiophene) (P3HT), at different *g* values. Here, $g = \left({\left\langle {d^2} \right\rangle /d_0^2 - 1} \right)^{1/2}$, where (*d* − *d*_0_) represents the fluctuations in interchain distance; *d* is the actual interchain distance; and *d*_0_ = 〈*d*〉 is the average interchain distance^14^. In order to determine the electronic band struture of P3HT, intensive DFT calculations are performed on a perfect crystal (*g* = 0%) (Fig. 1a, Supplementary Fig. 1); however, DFT becomes computationally challenging when mimicing the real structure of conducting polymers with intrinsic disorder. Instead, we proceed with a two-dimensional (2D) TB model, as shown in Fig. 1b, to mimic the behavior of a bulk polymer with a specific value of *g*. The average values of hopping parameters, *h* and *t* denoted for intrachain and interchain, respectively, are calculated for random samples whose interchain spacings possess realistic probability distribution functions (PDFs) as discussed below. The 2D TB model reproduces the DFT band dispersions very well for a perfect P3HT crystal (Fig. 1c), which allows us to perform further 2D TB calculations for P3HT with higher *g* values (Supplementary Fig. 2). In fact, to corroborate our methodology, after accounting for spatial correlations between the P3HT chains (therefore deviating away from a perfect crystal), we show that 2D TB reproduces the band dispersions obtained from DFT (Supplementary Fig. 3) as well. However, performing DFT on a randomly disordered sample with high paracrystallinity similar to that observed in experiments is not computationally tractable as it requires a very large number of molecules.

**Fig. 2:**
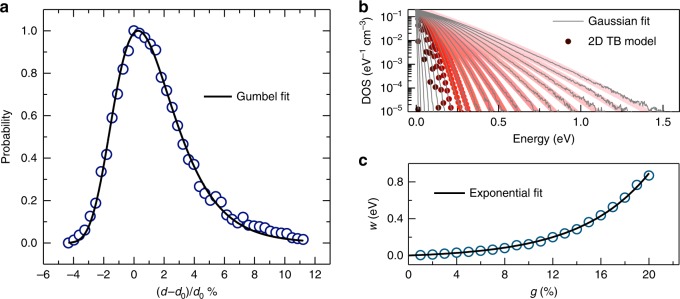
The relationship between the electronic density of states (DOS) and paracrystallinity (*g*). **a** Probability distribution function (PDF) as a function of the relative change in interchain distance for the case of $g = 7.93\%$, where PDF fits best with Gumbel function. **b** DOS tails calculated using a two-dimensional tight-binding (2D TB) model at different values of *g*, all structures can be fitted by Gaussian distribution. The case of *g* = 0% represents the highest occupied molecular orbital (HOMO) level of crystalline P3HT. **c** The width of DOS tail (*w*) as a function of *g*. *w* increases exponentially with *g*.

Therefore, in order to understand the effect of paracrystallinity on the electronic structure, we generated different configurations of P3HT, to introduce more positional disorder, using MD simulations (Supplementary Figs. 4–6 and 9, and Supplementary Note 1). It is found that all of these configurations result in PDFs that can be best fitted to a Gumbel distribution with different values of *w*, which are directly proportional to *g* (see Supplementary Figs. 7 and 10). For instance, the PDF of the interchain distance for *g* = 7.93% is shown in Fig. 2a. The origin of the specific shape of PDF arises from the asymmetric nature of Lennard–Jones potential (Supplementary Fig. 8), which demonstrates the fact that the interchain compressibility is harder than expansion. As a result, symmetric Gaussian distribution, as has been considered in other studies^2^, is not a good representation of the PDF. To study the electronic structure of P3HT for a wider range of (0−20%), a 2D TB model is then applied on crystallites composed of 100 π–π stacked chains, with 150 sites along the intrachain direction in each chain. This leads to a TB Hamiltonian with dimensions of 15,000 × 15,000. The relative change of interchain distance is generated based on the Gumbel distribution for a specific value of *g*. Figure 2b shows the electronic DOS tails for different *g* values. The DOS tails obtained from 2D TB model can be fitted by Gaussian functions whose *w* values increase exponentially with *g* (Fig. 2c). Thus, the electronic DOS tail is seen to be well represented by a Gaussian distribution, and the experimentally measured *g*, for instance, can be correlated to its width. On the other hand, other reports^2^ showed that by assuming a Gaussian function for the interchain spacings, an exponential tail of DOS can be generated by performing simple TB calculations, and therefore, this may alter the results of charge transport modeling. Therefore, choosing a proper PDF is a crucial step when modeling transport properties in disordered polymers.

### Electronic transport properties under Gaussian DOS

**Fig. 3:**
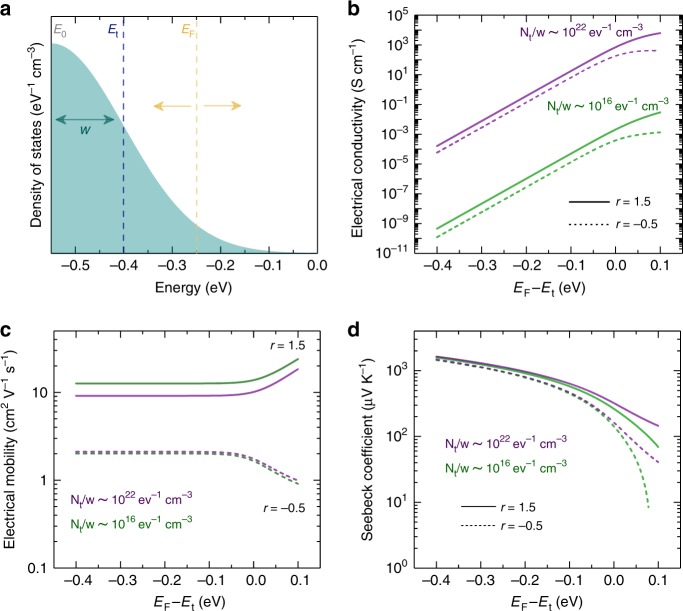
The parameters of Gaussian density of states (DOS) and transport properties. **a** Gaussian density of states of the band tail with broadening (*w*). The respective energy levels are Fermi energy (*E*_*F*_), transport energy (*E*_*t*_), and Gaussian peak (*E*_0_). For non-degenerate polymers, *E*_F_ is located at energies much lower than *E*_*t*_. **b** Electrical conductivity as a function of *E*_*F*_ − *E*_*t*_, where it strongly depends on the effective density of states, *N*_*t*_/*w*, and weakly on the scattering parameter, *r*. **c** Electrical mobility exhibits independent behavior with *E*_*F*_ − *E*_*t*_ when *E* ≪ *E*_*t*_, otherwise, it changes according to the scattering parameter, *r*. **d** Seebeck coefficient decreases with the absolute value of *E*_*F*_ − *E*_*t*_, and it is predominantly dependent on *r*. All values of *N*_*t*_/*w* and *r* are selected based on our analysis on different classes of conducting polymers as discussed below.

Armed with this confirmation of the shape of the DOS tail, we now use the framework of the Boltzmann transport equations to understand charge transport properties for a variety of polymers. Here, the electrical conductivity is given as^15^

1 $$\sigma = {\int} {\sigma _E} \left({ - \frac{{\partial f}}{{\partial E}}} \right){\mathrm{d}}E,$$

where *σ*_*E*_ for 3D isotropic systems under the relaxation time approximation is

2 $$\sigma _E = \frac{{2e^2}}{{3m^ \ast }}\tau \left(E \right)(E - E_{{t}})D\left(E \right)$$

and the DOS tail has a Gaussian shape given by

3 $$D\left(E \right) = \frac{{N_t}}{{w\sqrt {2\pi } }}e^{\frac{{ - \left({(E - E_{{t}}) - E_0} \right)^2}}{{2w^2}}},$$

where *e* is the electronic charge; m^*^ = *m*_*e*_ is the effective mass of charge carriers, which is approximated by the free electron mass (see Eq. (8)) without loss of generality; *τ* is relaxation time between two scattering events, and can be approximated as $\tau \left(E \right) = \tau _0\left({\frac{{E - E_t}}{{k_{\mathrm{B}}T}}} \right)^r$; *τ*_0_ is the relaxation time constant (10 fs, see Supplementary Note 2); *k*_B_ is Boltzmann constant; *T* is the absolute temperature; *E*_0_ is the energy at DOS peak (see Fig. 3a); *f* is Fermi-Dirac distribution function; *E*_t_ is the transport energy where charge carriers with lower energies cannot contribute to transport; *N*_t_/*w* is the ratio of the total energy states to the tail broadening (henceforth referred to as “effective DOS”).

The Seebeck coefficient is

4 $$S = \frac{1}{{eT\sigma }}\int\nolimits_0^\infty \tau \left(E \right)\,E\,D(E)\left({ - \frac{{\partial f}}{{\partial E}}} \right)(E - E_{{F}}){\mathrm{d}}E.$$

As doping increases, and *E*_*F*_ approaches to *E*_*t*_, *σ* increases as more states become available for carriers to occupy (Fig. 3b). Interestingly, *σ* increases also with lower degree of disorder (smaller *w*), irrespective of *r* value, which is in agreement with previously observed *σ* in conducting polymers^8^. Figure 3c shows the calculated Hall mobility (*μ*_Hall_) (Supplementary Eq. (7)) as a function of *E*_*F*_−*E*_*t*_ for different *r* values. Remarkably, *μ*_Hall_ is not very sensitive to the effective DOS. On the other hand, *S* decreases with *E*_*F*_−*E*_*t*_ and exhibits a prominent dependence on *r*, where higher *S* can be found for *r* = 1.5 (Fig. 3d). However, *S* is not sensitive to *w* (Supplementary Fig. 14). For thermoelectric applications, the power factor (*σS*^2^) is considered as a measure of the material’s electronic performance. Conducting polymers that have stronger energy-dependent scattering, *r* = 1.5, exhibit higher power factor than those that with *r* = −0.5 (Supplementary Fig. 15). Superior power factor, for polymers with *r* = 1.5, arises from the concurrent enhancement in *σ* and *S* compared with their counterparts with *r* = −0.5, for the same effective DOS. Therefore, our analysis corroborates in a rigorous manner that in order to improve the thermoelectric performance of conducting polymers, one would have to experimentally achieve highly doped yet perfectly ordered, highly packed polymers, where their charge carrier transport is described by *r* = 1.5.

### Modeling the experimental transport properties

**Fig. 4:**
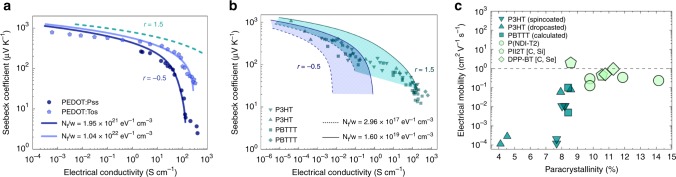
Charge transport properties for different classes of conducting polymers. Experimental values of Seebeck coefficient as a function of electrical conductivity for **a** poly(3,4-ethylenedioxythiophene):polystyrene sulfonate (PEDOT:PSS) (circles)^45^ and poly(3,4-ethylenedioxythiophene):tosylate (PEDOT:Tos) (pentagons)^8^, and for **b** poly(3-hexylthiophene) (P3HT) (triangles and circles) and poly(2,5-bis(3-tetradecylthiophen2-yl)thieno[3,2-b]thiophene) (PBTTT) (squares and diamonds)^46, 47^. The shaded stripes represent the calculated values using our model where the lower (dashed lines) and the upper (solid) limits are determined by the *N*_t_/*w* as specified. **c** Experimentally measured field-effect mobility as a function of paracrystallinity (*g*) for polymers with different molecular weights^1^. The conducting polymers are P3HT (downward triangles)^1^, P3HT (upward triangles)^24^, PBTTT (squares)^48^, poly(2,5-bis(3-tetradecylthiophen2-yl)thieno[3,2-b]thiophene) P(NDI-T2) (circles)^49^, Poly{[*N*,*N*-9-bis(2-octyldodecyl)naphthalene-1,4,5,8-bis(dicarboximide)-2,6-diyl]-alt-5,59-(2,29-bithiophene)}, [C, Si] (PII2T [C, Si]) (pentagons)^50^, and diketopyrrolopyrrole-benzothiadiazole copolymer, [C, Se] (DPP-BT [C, Se]) (diamonds)^51^. The dashed line is drawn at mobility of 1 cm^2^ V^−1^ s^−1^. The legend size is linked to the molecular weight (the larger molecular weight, the larger the legend).

Following this, we study in detail the relationship between *S* and *σ*, for three prototypical conducting polymers, PEDOT, P3HT, and PBTTT (Fig. 4a, b). PEDOT-based polymers are some of the most studied, where their conjugated backbones form linear chains, which can be separated by networks of other organic materials such as PSS or Tos. We find that PEDOT-based polymers are best modeled when *r* = −0.5, while the model deviates away from experimental observations significantly in the case of *r* = 1.5, which is consistent with previous reports where 3D transport was assumed^6^. To interpret this distinct *r* value obtained for PEDOT-based polymers, it is interesting to examine the basic structure of PEDOT chain which has a linear form of EDOT monomers, which lacks any side chains that promote backbone rigidity, hence more chain vibrations (phonons) would exist in PEDOT-like polymers. Interestingly, PEDOT:Tos exhibits less energy broadening (*w* ~ 0.2 eV), and hence larger *N*_t_/*w*, compared to PEDOT:PSS (*w* ~ 0.8 eV), indicating that PEDOT:Tos chains tend to be more aligned, which is supported by grazing incident wide angle X-ray scattering (GIWAXS)^8^. In fact, a large broadening of DOS tail (~1 eV) has been reported in highly disordered PEDOT:PSS thin films using ultraviolet photoelectric spectroscopy measurements^16^, which agrees well with our fitted values of *w* for PEDOT:PSS. Several experiments were able to successfully align PEDOT, and hence enhance its electrical conductivity^17,18^.

On the other hand, P3HT and PBTTT possess a distinct monomer structure with additional side chains, which help in enhancing the electronic coupling in the π−π stacking direction by facilitating the backbones to be aligned in 2D planes^19^. Usually, upon doping P3HT and PBTTT, the ionized counterions are preferentially positioned and arranged in between the side chains^20^ that essentially enhance the backbone rigidity. These dissimilar chemical features in P3HT or PBTTT, compared to PEDOT-like polymers, would explain why charge carriers in this family of polymers would possess a different scattering mechanism. Indeed, it is found that P3HT and PBTTT exhibit *r* = 1.5 (Fig. 4b), suggesting that the dominant scattering mechanism is determined by the ionized counterions. Interestingly, the relaxation time calculations for charge carriers in P3HT at moderate doping levels (*n* ~ 10^20^ cm^−3^) show that ionized impurity scattering exhibits shorter relaxation times compared to scattering from acoustic phonons (see Supplementary Note 2). Moreover, since P3HT and PBTTT have conducting paths only along their backbones, rather than the insulating side chains, they have less energy states per unit volume (smaller *N*_t_), and, hence, their effective DOS (*N*_t_/*w*) is smaller compared to that for PEDOT-based polymers, regardless of *w* value. Indeed, the fitting bands overlap with the experimental values of P3HT and PBTTT at lower values of *N*_t_/*w* (10^17^–10^19^ eV^−1^ cm^−3^) compared to *N*_t_/*w* of PEDOT (~10^21^ eV^−1^ cm^−3^). The effective DOS of P3HT and PBTTT conducting polymers with side chains are found to be five orders of magnitude lower than the one for PEDOT polymers. *N*_t_ can increase upon doping, which explains the larger *N*_t_/*w* values at higher range of electrical conductivities (Fig. 4b)^16^. Similar order-of-magnitude difference in the transport coefficient (*σ*_*E*0_) was also observed by Kang and Snyder^6^ when comparing PEDOT with other polymers that have side chains. We consequently explain that the distinct features of monomer structures of those two classes of conducting polymers result in different values of *N*_t_/*w*. Another interesting finding in P3HT and PBTTT is that *w* values are smaller (0.1−0.3 eV) than the ones found for PEDOT:PSS (*w* ~ 0.8 eV), which is consistent with the fact that polymers with side chains tend to aggregate in crystallized domains, and hence narrower DOS tails could be obtained. It is worth mentioning that, in our analysis, *E*_F_ exceeds *E*_t_ at most by ~0.4 eV, which falls in the same range as DOS broadening, which is physically reasonable^21,22^. Furthermore, the fitting curves align well with the experimental results of PEDOT:PSS as *σ* > 1 S/cm, while they deviate from the ones of PEDOT:Tos at *σ* < 0.3 S/cm (Fig. 4a), which may be attributed to a different transport nature such as hopping as previously reported^5^. Similarly, in Fig. 4b, there are at least three orders of magnitude where *σ* > 0.1 S/cm at which transport can be described well by Boltzmann transport equations. Note that for highly doped conducting P3HT and PBTTT (*σ* > 100 S cm^−1^), bipolarons (spinless charge carriers) can be formed within the polymer backbones^23^, which then possibly cause a deviation from Fermi-Dirac statistics.

Next, we extend our discussion to charge mobility (*μ*) of carriers occupying the Gaussian DOS tail. In conducting polymers, the increase in *μ* with molecular weight is well known^24,25^, but its dependence on *g* is still conceptually lacking^1^. We correlate their field-effect transistor (FET) mobility (*μ*_FET_) with the experimentally measured *g* (Fig. 4c). Strikingly, *μ*_FET_ does not show any clear dependence on *g* opposite to what is commonly stated that molecular ordering enhances *μ*_FET_ (Supplementary Fig. 13). This shows that *μ*_FET_ depends only on the degree of polymerization; as the molecular weight increases and saturates, so does the *μ*_FET_. In fact, *μ*_FET_ of some highly disordered (*g* > 10%) high-molecular weight conducting polymers, such as P(NDI-T2), PII2T[C, Si], and DPP-BT[C, Se], exceeds the ones that have been reported for more crystalline polymers, such as P3HT and PBTTT (Fig. 4c). The highly disordered, “near amorphous” polymers tend to form short range crystalline aggregates, which are electrically interconnected by “tie-chains”, and hence allowing for high *μ*_FET_ despite their high *g* values^1^.

Finally, we utilize BTE formalism to understand Hall mobility (*μ*_Hall_) (Supplementary Eq. (7) and Supplementary Note 3), which represents the intrinsic *μ*_Hall_ in the bulk semiconducting polymer, unlike FET geometry where *μ*_FET_ reflects the transport at the semiconductor-dielectric interface. Although *μ*_*Hall*_ is difficult to measure for most paracrystalline conducting polymers, a few reports^20,26^ have attempted this measurement albeit by considering the degenerate form of mobility (*μ*_Hall_ = *σ*/*ne*) without taking into account *r* (Supplementary Fig. 12). For instance, Kang et al.^20^ measured *μ*_Hall_ in highly doped PBTTT and PEDOT:PSS. At such high levels of doping, our model predicts accurately *μ*_Hall_ of PBTTT if we consider *r* = 0.5 (Supplementary Table 2), different from *r* = 1.5 as deduced from *S* – *σ* relationship (Fig. 4b). In highly doped PBTTT, the ionized anions which reside in the side chain regions are partially screened by the large concentration of charge carriers that travel in the π–π stacking^20,27^, and hence, in addition to ionized impurities, acoustic phonons will contribute to the total scattering mechanism, which explains the different value of *r*. On the other hand, PEDOT:PSS exhibits highly degenerate features; hence, *R*_H_ does not depend on *r* (Supplementary Fig. 11). Therefore, one can use $\mu _{\mathrm{{Hall}}} = \sigma \,R_{\mathrm{H}} = \frac{\sigma }{{ne}}$ to calculate mobility which indeed results in a good agreement between experimental and calculated values (Supplementary Table 2). This solidifies our claim that *μ*_Hall_ is predominantly dominated by *r* and not the *N*_t_/*w* ratio at a particular doping level. Our findings validate generally accepted design principles for organic-based electronics, where *r* = 1.5 polymers are preferred as they can potentially exhibit higher intrinsic mobilities, potentially greater than 10 cm^2^ V^−1^ s^−1^ (Fig. 2c). Additionally, monomers that enhance the three-dimensional molecular packing (larger *N*_t_) along with improved chain alignment (smaller *w*) can enhance electrical conductivity, without Seebeck saturation until much higher doping levels.

## Discussion

In summary, we provide a general framework to understand charge transport in conducting polymers by solving the full Boltzmann transport equation. TB model calculations supported by DFT and MD reveal that the DOS tail in conducting polymers can be well described by a Gaussian distribution which its width increases exponentially with paracrystallinity. By correlating experimental values of Seebeck coefficient and electrical conductivity for different classes of conducting polymers, we find that charge transport can be fully described by the scattering parameter, and the ratio of the total number of energy states to DOS broadening. In addition, our framework explains well experimentally observed values of field-effect and Hall mobilities in this class of materials. Our study sheds light on the basic physics of charge transport in conducting polymers that will help in designing better conducting polymers with desired functionalities in many electronic applications.

## Methods

### DFT calculations

The initial structure of P3HT crystal was constructed with lattice parameters^28,29^: *a* = 16.18 Å, *b* = 7.72 Å, *c* = 7.89 Å, *α* = *β* = 90° and *γ* = 86.16° that are in agreement with the experimental parameters reported previously. Atomic positions were optimized by using Perdew, Burke and Ernzerhof (PBE)^30^ exchange-correlation functional with Tkatchenko–Scheffler (TS) semi-empirical dispersion correction parameters^31^ in CASTEP plane-wave pseudopotential software package^32^. Ultrasoft pseudopotentials were adopted with 410 eV cut-off energy. Self-consistent field (SCF) convergence criteria are set to 2.0 × 10^−6^ eV/atom. During optimization, 1.0 × 10^−5^ eV/atom for energy, 0.001 Å for max distance, and 0.03 eV/Å for maximum force cut-off parameters were applied to get fully relaxed structures. Interchain distance for equilibrium structure of P3HT was 3.862 Å (see Fig. 1a). Then, the interchain distance was adjusted manually between 2.862 and 5.862 Å (from *d* − 1 to *d* + 2 Å) to calculate the band structure. We calculated five different characteristic parameters of the band structures which are intrachain conduction band width (*W*_c−h_), intrachain valence band width (*W*_v−h_), interchain conduction band with (*W*_c_), interchain valence band with (*W*_v_), and band gap (*E*_g_) as represented in Supplementary Fig. 1a. The band structure parameters calculated for different interchain distances are given in Supplementary Fig. 1b. *E*_g_ and *W*_v−h_ increase and *W*_c_, *W*_v_, and *W*_c−h_ decrease with increasing interchain distance. All the parameters can be well fitted by exponential functions. Next we will construct a TB model from the band structure parameters. In order to further validate our approach, we have done more DFT and TB calculations at certain values of disorder. In a cell with two P3HT chains, we can move one chain against another by *δ*, and this will cause the final separation to be (*d*_0_ + *δ*) and (*d*_0_ −  *δ*). We calculated the band structures using both PBE DFT and our TB model (Supplementary Fig. 2).

### TB calculations

The electronic structure simulation of a polymer supercell with large paracrystallinity (*g*) using DFT is computationally challenging. Instead, we develop a two-dimensional (2D) TB model to mimic the behavior of a polymer bulk for specific value of *g*. Here, *g* is defined as $g = ((\delta /d_0)^2)^{0.5} = (\langle d^2\rangle /d_0^2 - 1)^{0.5}$, where *δ* = *d* − *d*_0_ represents the change of interchain distance between the backbones of the adjacent chains, *d* is the interchain distance, and *d*_0_ = 〈*d*〉 is the averaged interchain distance^14^. The average 〈*d*^2^〉 is done for randomized samples generated according to realistic distribution functions (see below).

Based on P3HT geometry, there are only two directions for charge transport. One direction is along the polymer backbone and the electron hopping is caused by the delocalized π-orbital. Another direction is along the π − π stacking direction (interchain transport). We adopt one *p*-like orbital per monomer, and thus the interaction between neighboring sites (intrachain) is *h* = *t*_*ppπ*_, and the interchain interaction in the π − π stacking direction is *t*  =  *t*_*ppσ*_.

It is straightforward to write the Hamiltonian for a perfect two-dimensional lattice:

5 $$H = \left[ {\begin{array}{*{20}{c}} {h{\mathrm{e}}^{ik_x\pi } + h{\mathrm{e}}^{ - ik_x\pi }} & {t{\mathrm{e}}^{ik_y\pi } + t{\mathrm{e}}^{ - ik_y\pi }} \\ {t{\mathrm{e}}^{ik_y\pi } + t{\mathrm{e}}^{ - ik_y\pi }} & {h{\mathrm{e}}^{ik_x\pi } + h {\mathrm{e}}^{ - ik_x\pi }} \end{array}} \right] = \left[ {\begin{array}{*{20}{c}} {2h\cos k_x\pi } & {2t\cos k_y\pi } \\ {2t\cos k_y\pi } & {2h\cos k_x\pi } \end{array}} \right].$$

Solving the eigen problem and we can obtain the eigen energies as *E*(*k*_*x*_, *k*_*y*_) = 2*h* cos *k*_*x*_*π* ±  2*t* cos *k*_*y*_*π*. Note that the hopping parameters, *h* and *t*, depend on the interchain distance *d*; therefore, it is possible to fit the parameters using the band widths obtained from DFT calculation. So, one can find that *W*_c_ = 4*t*_c_, *W*_v_ = −4*t*_v_, *W*_c−h_ = 2*h*_c_, and *W*_v−h_ = −2*h*_v_. Here we assume $t\left(\delta \right) = \alpha _t \cdot \exp \left({ - \beta _t \cdot \delta } \right)$ and $h\left(\delta \right) = h_0 + \alpha _h \cdot \exp \left({ - \beta _h \cdot \delta } \right)$, where *δ* = *d* − *d*_0_ is the change in interchain distance. These relations ensure that the interchain hopping decays to zero when the interchain distance approaches infinity, and the intrachain hopping converges to finite value simultaneously.

After fitting, we find $t_{\mathrm{c}}\left(\delta \right) = 0.1458\exp \left({ - 1.5900\,\delta } \right)$ and $h_{\mathrm{c}}\left(\delta \right) = 0.7934 + 0.0153\exp \left({ - 1.5599\,\delta } \right)$ for conduction bands, and $t_{\mathrm{v}}\left(\delta \right) = - 0.1495\exp \left({ - 1.7081\,\delta } \right)$ and $h_{\mathrm{v}}\left(\delta \right) = - 0.9907 + 0.0628\exp \left({ - 1.6033\,\delta } \right)$ for valence bands. The unit of all the hopping parameters is eV, and the unit of *δ* is Å. Interestingly, the 2D TB model reproduces the DFT band dispersions very well (Supplementary Figs. 2 and 3).

From the band dispersions of P3HT crystal, it is possible to find the electronic effective masses in interchain and intrachain directions for both valence and conduction bands as the following:

6 $$E =\frac{{{\hbar} ^2 \, k^2}}{{2\,m {\ast} }} = \frac{{{\hbar} ^2 \, \tilde k^2}}{{2 \, m {\ast} }}\left({\frac{{2\pi }}{L}} \right)$$

7 $$E\left({\vec k} \right) = 2t_0\cos k_y\pi \pm 2h_0\cos k_x\pi,$$

8 $$\left| {m_x \ast } \right| = \frac{{2\hbar ^2}}{{a^2}}\frac{1}{{h_0}},\left| {m_y \ast } \right| = \frac{{2\hbar ^2}}{{l^2}}\frac{1}{{t_0}}.$$

If we take the interchain and intrachain distances to be *a* = 3.862 Å and *l* = 3.945 Å, respectively, the effective masses will be $\left({m \ast } \right)_{\mathrm{{inter}}}^{\mathrm{c}} = 7.007m_e,\,\left({m \ast } \right)_{\mathrm{{inter}}}^{\mathrm{v}} = 6.835\,m_e,$ $\left({m \ast } \right)_{\mathrm{{intra}}}^{\mathrm{c}} = 1.211m_e,\,\left({m \ast } \right)_{\mathrm{{intra}}}^{\mathrm{v}} = 1.055m_e$.

The total effective mass (m*) accounts for both transport directions; interchain $\left({m_{\mathrm{{inter}}} \ast } \right)$ and intrachain $\left({m_{{\mathop{\rm{intra}}}} \ast } \right)$:

9 $$\frac{1}{{m \ast }} = \frac{1}{{m_{\mathrm{{inter}}} \ast }} + \frac{1}{{m_{\mathrm{{intra}}} \ast }}$$

so, if we take the calculated values of $m_{\mathrm{{inter}}} \ast$ and $m_{\mathrm{{intra}}} \ast$ for P3HT obtained from the TB model, the total m* is found to be close to the rest mass of electron (*m*_0_). A lower m* is found in the intrachain direction for other polymers as reported previously^33^.

### MD simulations

Three different calculations were performed based on three distinct molecular arrangements (Supplementary Figs. 4–6) of P3HT to create a range of *g* values by using classical simulation methods based on COMPASS force field^34^. NVT ensemble, Nose Thermostat, and 1 fs step size were used in all simulations. For the three different simulations, the *g* values were calculated to be (i) 1.04%, (ii) 2.40%, and (iii) 7.93%. Probability distribution functions (PDF) for the interchain distance of these simulations are given in Supplementary Fig. 7. These results show that the temperature effect can only cause a relatively low paracrystallinity (*g* < 8%) in P3HT crystal. However, it should be noted that we used optimized well-packed crystal under periodic boundary conditions as an initial structure.

In order to get an analytic expression of the Probability distribution functions (PDF) so that we can generate a distribution function for any *g*, we attempted four different PDFs, namely Gumbel, Rayleigh, Logistic, and Gaussian distributions, to fit the PDF we found from the MD simulations (Supplementary Fig. 7). It can be found that the Gumbel distribution fits best compared to the other three distributions, especially when *g* is large. Therefore, Gumbel distribution is considered in our calculations later when generating a wider range of paracrystallinity (0 < *g* < 20%).

The PDF of Gumbel distribution can be written as

10 $$f\left(x \right) = \frac{1}{\beta }\exp \left[ { - \left({z + e^{ - z}} \right)} \right],$$

where $z = \frac{{x \, - \, \mu }}{\beta }$, and *μ* and *β* are fitting parameters. In our calculations, *μ* is set to 0 so that the peak of PDF always is at 0. Meanwhile, since the standard deviation of Gumbel distribution is $\pi \beta /\sqrt 6$, which should equal to *g*; therefore, for a given *g*, the PDF of the relative change of interchain distance *δ*/*d*_0_ can be described by a Gumbel distribution with $\beta = \sqrt 6 g/\pi$.

All the PDFs possess similar asymmetric shape. The origin of this asymmetry comes from the asymmetric Lennard–Jones potential, which describes harder interchain compressibility than interchain expansion. The relation for the total energy with interchain distance (given as “*d*_0_” in Supplementary Fig. 1b) is calculated by molecular mechanics calculations and is plotted in Supplementary Fig. 8. As a result, symmetric Gaussian distribution does not provide a good fitting to the PDF for P3HT interchain distances.

It is worth mentioning that, in our work, we neglect the possibility of any change in the intrachain distance between neighboring sites, which does not affect our overall calculations of charge transport. We have two reasons that justify this approximation. First, the intrachain connection is covalent bonds, which is much stiffer than the nonbonding interactions among the chains (van der Waals), which means that the disorder is less likely to take place in the intrachain direction. Second, the intrachain hopping parameter is much larger than the interchain one, so one can expect that intrachain disorder mainly results in deeper energy states in the DOS.

### Relaxation time calculations

The electronic structure calculations were performed by the projector augmented wave^35^ method with PBE exchange-correlation functional^36^ in Vienna Ab initio Simulation Package (VASP)^37^. The structural optimization was conducted by PBE functional with the dDsC dispersion correction^38^. Throughout the calculations, the convergence criterion of the total energy was set to be 10^−5^ eV in the SCE iteration. The cut-off energy for the plane-wave basis set was set to be 600 eV. The cut-off radius for pair interactions was set to be 50 Å. The *k*-mesh of 2 × 1 × 2 was used during the structural optimization. The single-point energy and charge density calculations were performed on the *k*-mesh of 4 × 2 × 4. For relaxation time calculations, a dense Monkhorst-Pack *k*-mesh of 40 × 8 × 40 was used, which amounts to a total number of 12,800 points in the irreducible Brillouin zone.

According to the Fermi’s Golden rule^39^, the relaxation time, *τ*_*k*_, takes the form

11 $$\frac{1}{{\tau _{\boldsymbol{k}}}} = \left({\frac{{2\pi }}{{\hbar N_{\boldsymbol{k}}{\mathrm{\Omega }}}}} \right)\mathop {\sum }\limits_{{\boldsymbol{k}}^{\prime} } \left| {M\left({{\boldsymbol{k}},{\boldsymbol{k}}^{\prime} } \right)} \right|^2{\updelta}\left({\varepsilon _{\boldsymbol{k}} - \varepsilon _{{\boldsymbol{k}}^{\prime} }} \right)\left({1 - \cos \theta } \right),$$

where *ħ* is the reduced Planck constant; *N*_*k*_ is the number of *k*-points; Ω is the unit cell volume; *δ*(*ε*_***k***_ − *ε*_***k***′_) is Dirac delta function to ensure the energy conservation for elastic scattering events; *θ* is the scattering angle between the states ***k*** and ***k***′; the summation runs over all available final states. Herein, the acoustic phonon scattering was modeled by the deformation potential (DP) theory^40^, under which the scattering matrix element can be expressed as

12 $$\left| {M({\boldsymbol{k}},{\boldsymbol{k}}^{\prime} )} \right|^2 = \frac{{k_{\mathrm{B}}TE_1^2}}{{C_{ii}}},$$

where *k*_B_ is Boltzmann constant; *E*_1_ is the DP constant, and *C*_*ii*_ (*ii* = *aa*, *bb* and *cc*) is the elastic constant (Supplementary Table 1). Both the DP constant and elastic constant were evaluated from first-principles calculations. The elastic constant was calculated by stretching the unit cell along the crystal axes *a*, *b*, and *c* directions separately by ±0.5% and ±1.0%, and then fitting the total energy, *E*, of deformed lattice with respect to the dilation, Δ*l*/*l*_0_ via the formula, $(E - E_0)/{\mathrm{\Omega }}_0 = C_{ii}(\Delta l/l_0)^2/2$, where *E*_0_ and *l*_0_ are the total energy and lattice parameter at equilibrium, respectively; Δ*l* is the change of lattice parameters. To evaluate the hole DP constant, we calculated the band energies with the lattice deformed, and then fit the valence band maximum (VBM) to the dilation, Δ*l*/*l*_0_ via the formula, $E_1 = \Delta E_{{\mathrm{VBM}}}/(\Delta l/l_0)$, where, Δ*E*_VBM_ is the position change of VBM with the lattice deformation. The lowest energy level was assumed to be the energy reference point^41^.

The Brooks–Herring approach^42^ was adopted to model the screened Coulomb scattering caused by the ionized impurities, under which the scattering matrix element has the form

13 $$\left| {M({\boldsymbol{k}},{\boldsymbol{k}}^{\prime} )} \right|^2 = \frac{{n_{\mathrm{I}}\left({q_{\mathrm{I}}e} \right)^2}}{{{\mathrm{\Omega }}\left({\varepsilon _{\mathrm{r}}\varepsilon _0} \right)^2\left({L_{\mathrm{D}}^{ - 2} + \left| {{\boldsymbol{k}}^{\prime} - {\boldsymbol{k}}} \right|^2} \right)^2}},$$

where *n*_I_ is the randomly located scattering centers per unit cell; *q*_I_ is the charge of impurities; *e* is the elementary charge; $L_{\mathrm{D}} = \sqrt {\varepsilon _{\mathrm{r}}\varepsilon _0k_{\mathrm{B}}T/(e^2N_{\mathrm{h}})}$ is the Debye screening length; *N*_h_ is the hole concentration; *ε*_r_ is the relative dielectric constants of a material, and *ε*_0_ is the dielectric constants of vacuum (≈8.85 × 10^−12^ C^2^ N^−1^ m^−2^). The relative dielectric constants of P3HT was set to be 3.50 (ref. ^43^). The relaxation times were derived from the revised BoltzTraP code^44^.

The acoustic phonon and ionized impurity scattering times are 203 and 106 fs, respectively, at hole concentration of 10^20^ cm^−3^ at room temperature. So the impurity scattering plays a dominant role for P3HT.

## Supplementary information

Supplementary Information

## Data Availability

Any of the data used generated via simulations can be provided by the authors upon email request to the corresponding authors.

## Code availability

Any of the code that we have used for our conclusions can be provided by the authors upon email request to the corresponding authors. Basic parts of the MATLAB code is shown in Supplementary Note 4.
