# Supplementary material for: Correlating charge and thermoelectric transport to paracrystallinity in conducting polymers
Source: Nat Commun. 2020 Apr 8;11:1737. doi: 10.1038/s41467-020-15399-2 (PMC7142092; doi:10.1038/s41467-020-15399-2)
Supplement: Supplementary file 1 — Supplementary Information [file 41467_2020_15399_MOESM1_ESM.pdf]

Supplementary Information

Correlating charge and thermoelectric transport to paracrystallinity in conducting polymers

Anas Abutaha[1#], Pawan Kumar[1#], Erol Yildirim[2,3#], Wen Shi[2], Shuo-Wang Yang[2], Gang Wu[2], and Kedar Hippalgaonkar[1*]*

[1] Institute of Materials Research and Engineering, Agency for Science Technology and Research, #08-03, 2 Fusionopolis Way, Innovis, Singapore 138634
[2] Institute of High Performance Computing, Agency for Science, Technology and Research, 1 Fusionopolis Way, #16-16 Connexis, Singapore 138632
[3] Department of Chemistry, Middle East Technical University, 06800 Ankara, Turkey
[4] Materials Science and Engineering, Nanyang Technological University, 50 Nanyang Avenue, Singapore 639798, Singapore

# these authors contributed equally to this work
*Corresponding authors

Contents

Supplementary Figures

Supplementary Tables

Supplementary Notes

Supplementary References

Supplementary Figures

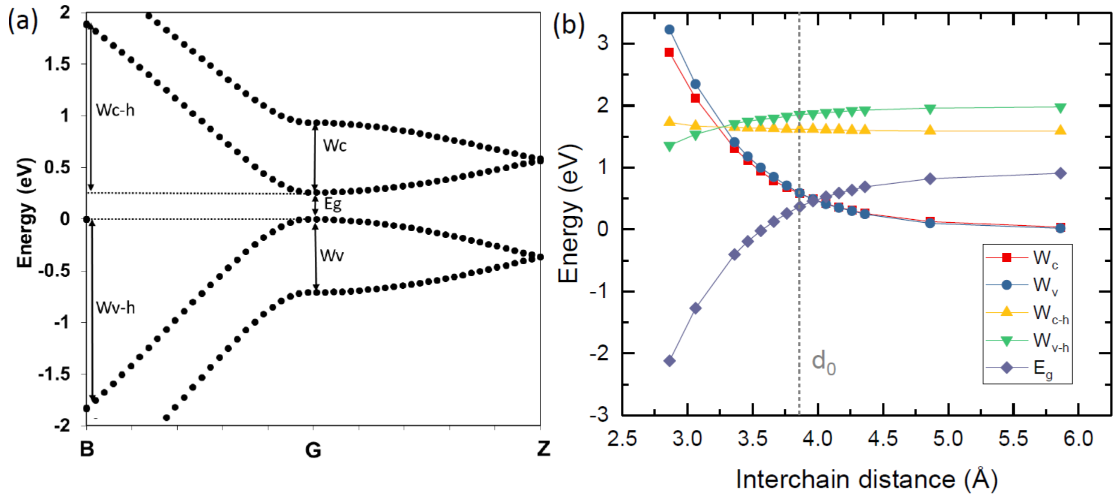

Supplementary Figure 1. Density Functional Theory (DFT) Calculations. (a) Calculation of initial parameters for tight binding model from band structure. (b) Intrachain conduction band width (W_c), intrachain valence band width (W_v), interchain conduction band with (W_{c-h}), interchain valence band with (W_{v-h}) and band gap (E_g) for different interchain distances.

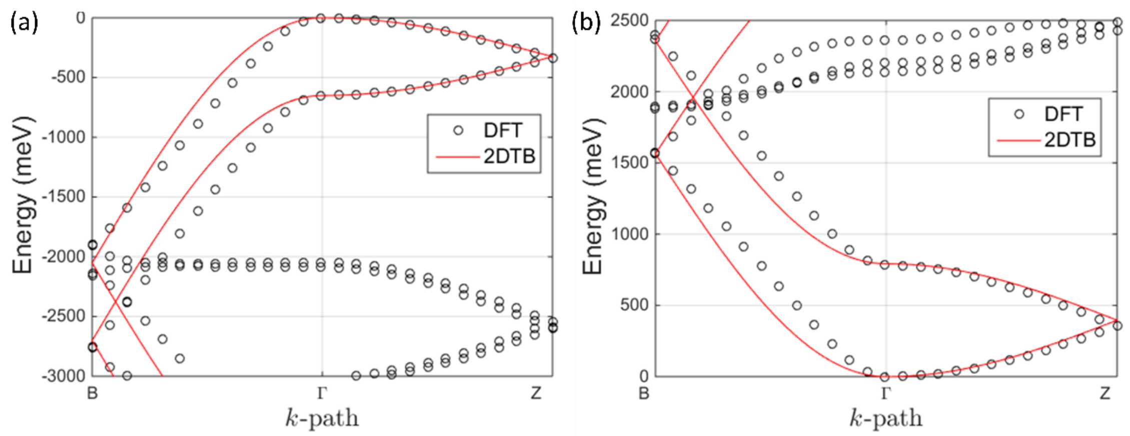

Supplementary Figure 2. **The electronic band structures obtained from DFT calculations and 2D TB model.** Band structures for (a) valence and (b) conduction bands.

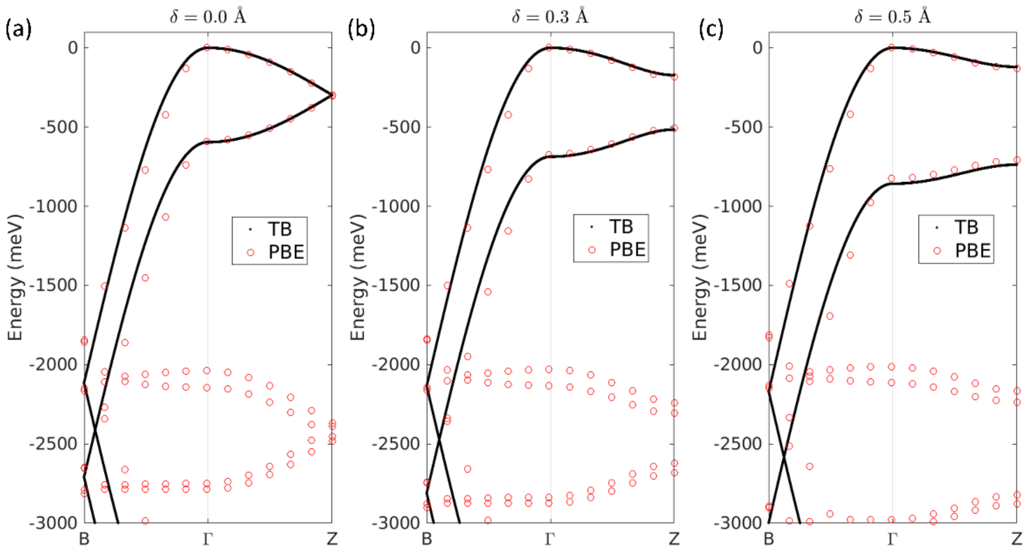

Supplementary Figure 3. The Effect of chain displacement on the electronic structure of P3HT. DFT (circles) and TB (line) electronic band structures for two chains of P3HT where one chain is displaced by (a) δ=0, (b) 0.3, and (c) 0.5 Å. As shown in Supplementary Fig. 3, the tight-binding model can reproduce the PBE DFT band structures of the systems with spatial correlations (δ=0.3 and 0.5 Å).

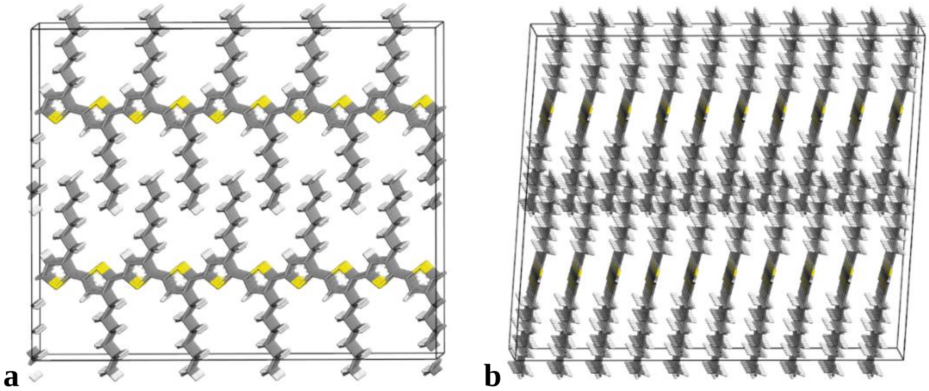

Supplementary Figure 4. Interdigitated configuration of P3HT. Supercell of 2×5×5 with (a) *xy* and (b) *yz* planes.

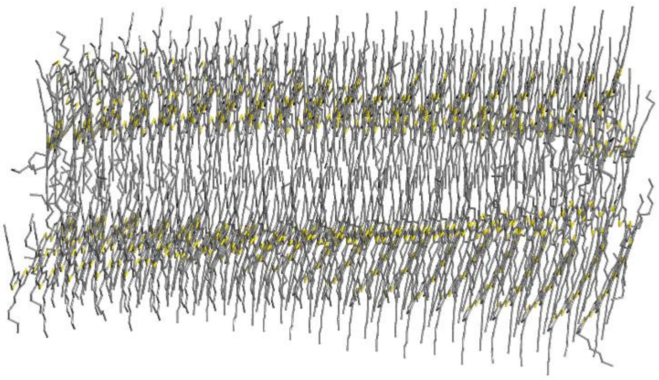

Supplementary Figure 5. Non-periodic configuration of P3HT. supercell of 2×10×9 with 720 monomers.

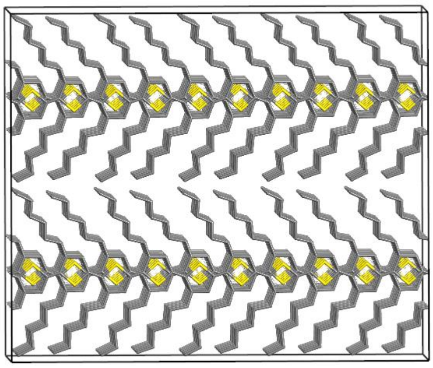

Supplementary Figure 6. Fishbone-like configuration of P3HT. supercell of 2×5×5.

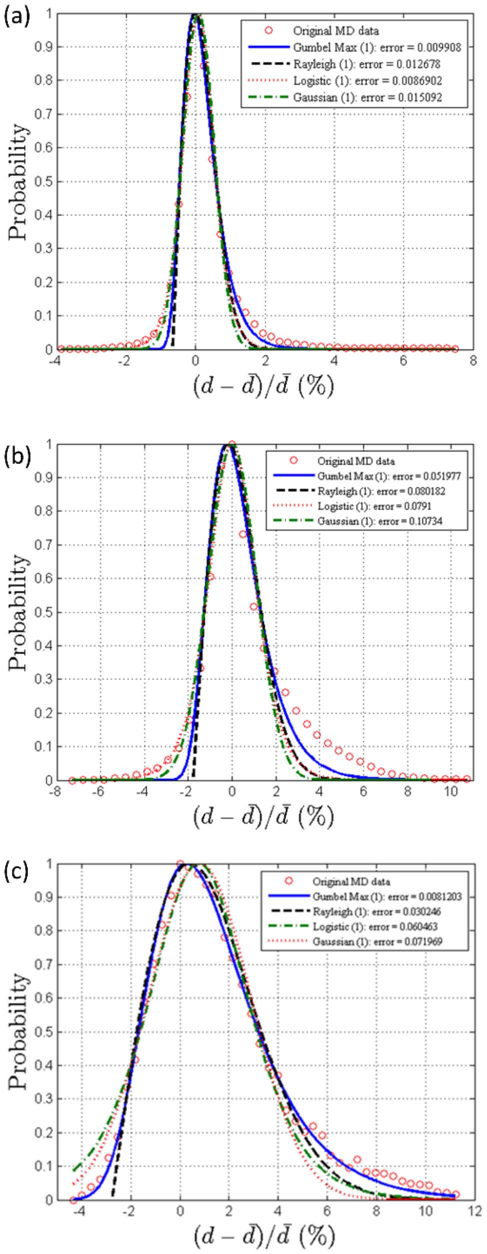

Supplementary Figure 7. Probability distribution function (PDF) of the relative change of interchain distance. PDF at g values of (a) 1.04%, (b) 2.40% and (c) 7.93%.

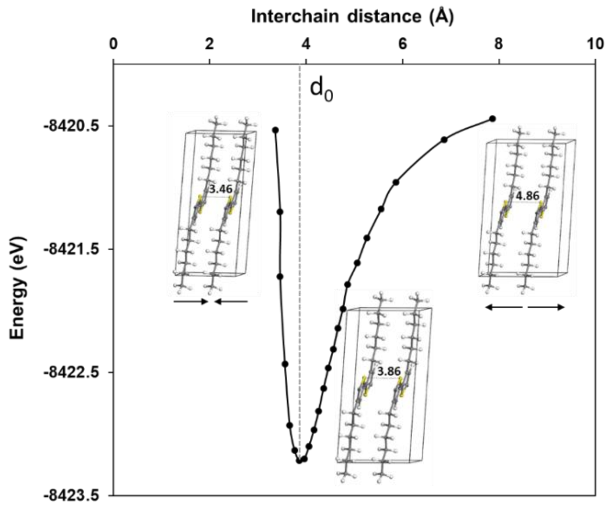

Supplementary Figure 8. Lennard-Jones potential. System energy (eV) as a function of interchain distance (Å) for P3HT crystal.

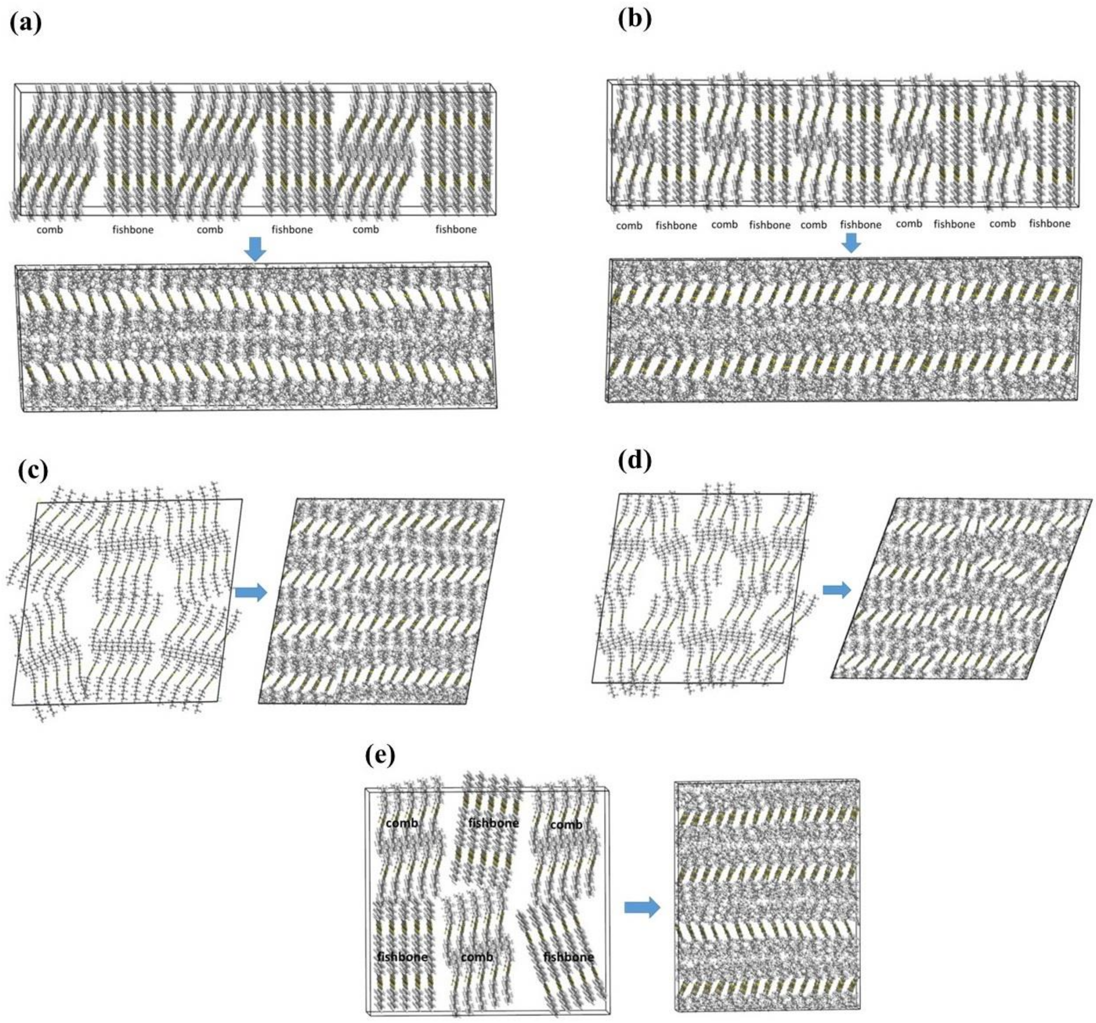

Supplementary Figure 9. **Mixing configurations of P3HT before and after annealing**. All the initial configurations contain randomly packed crystallites: (a) three comb-like regions and three fishbone-like regions, and in each region five chains exist, (b) five comb-like regions and five fishbone-like regions, and in each region three chains exist, (c) six comb-like regions, and

in each region five chains exist. (d) ten comb-like regions, and in each region three chains exist, (e) three comb regions and three fishbone regions, and in each region five chains exist.

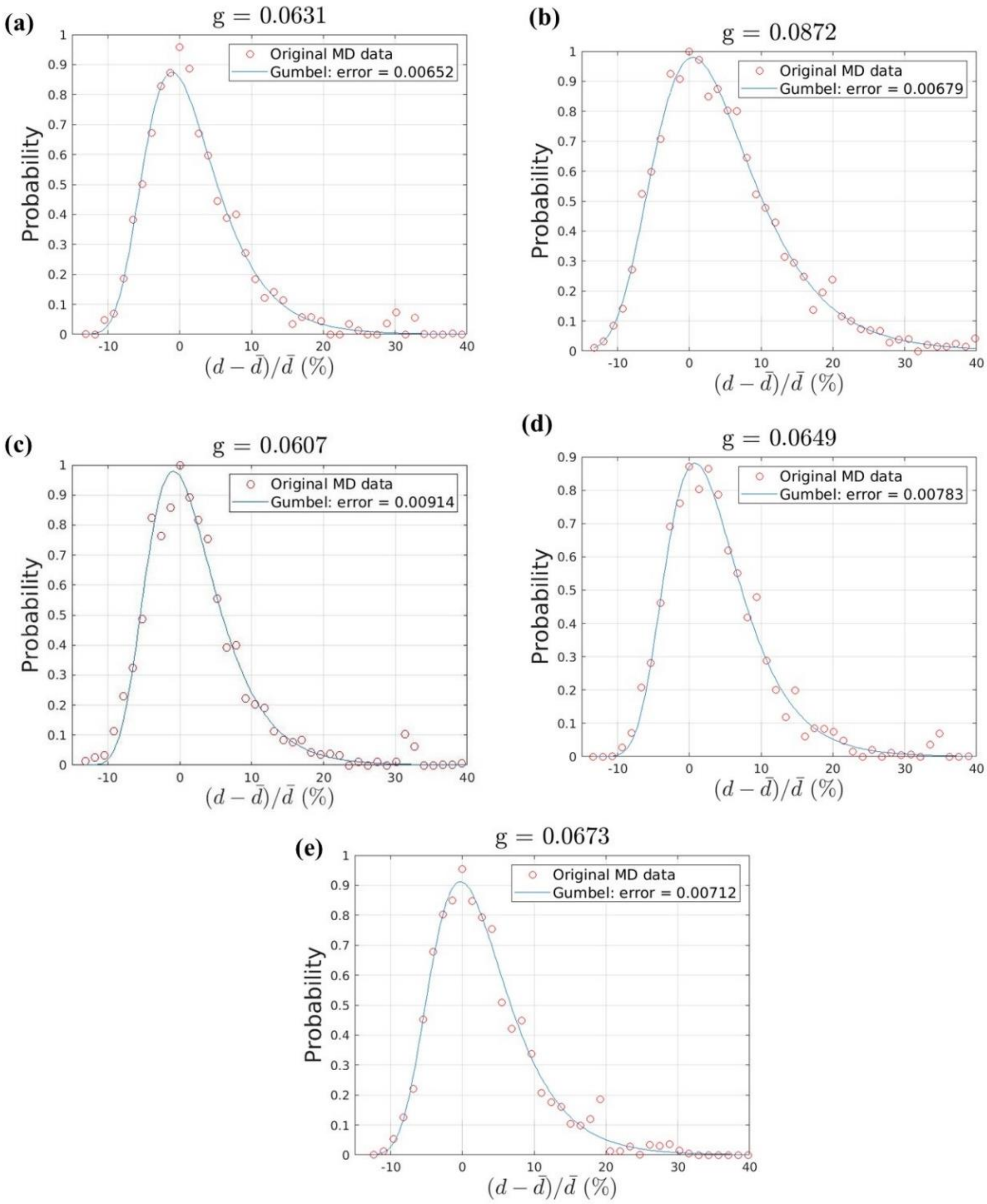

Supplementary Figure 10. Interchain distance distributions for the same cases in Supplementary Figure 9. Interchain distance distributions can be best fitted by Gumbel function for paracrystallinity (g) values of (a) 6.31 %, (b) 8.72 %, (c) 6.07 %, (d) 6.49 %, (e) 6.73 %.

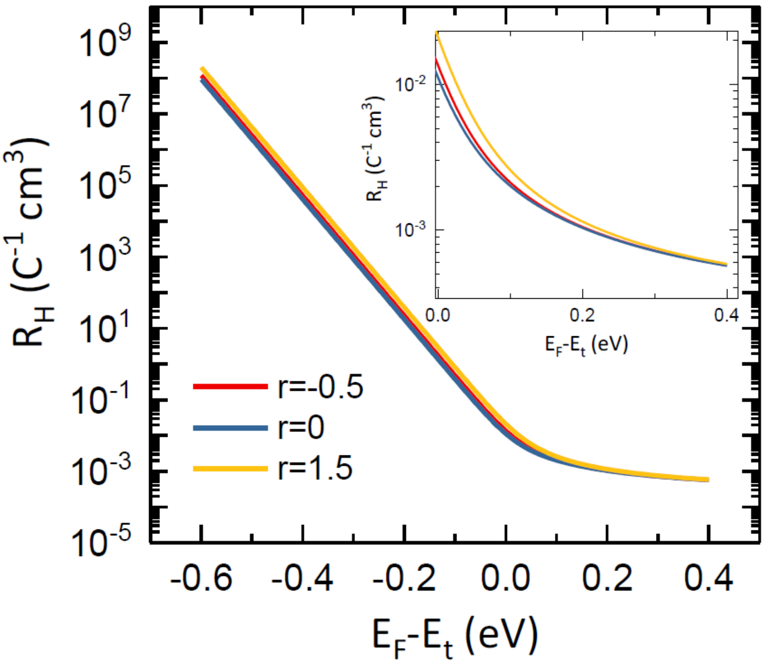

Supplementary Figure 11. Calculated Hall coefficient (R_H). R_H is calculated by taking $D(E)$ to be Gaussian, as a function of $E_F - E_t$ for different r values. The inset shows that R_H converges for all scattering parameters in high doping regime.

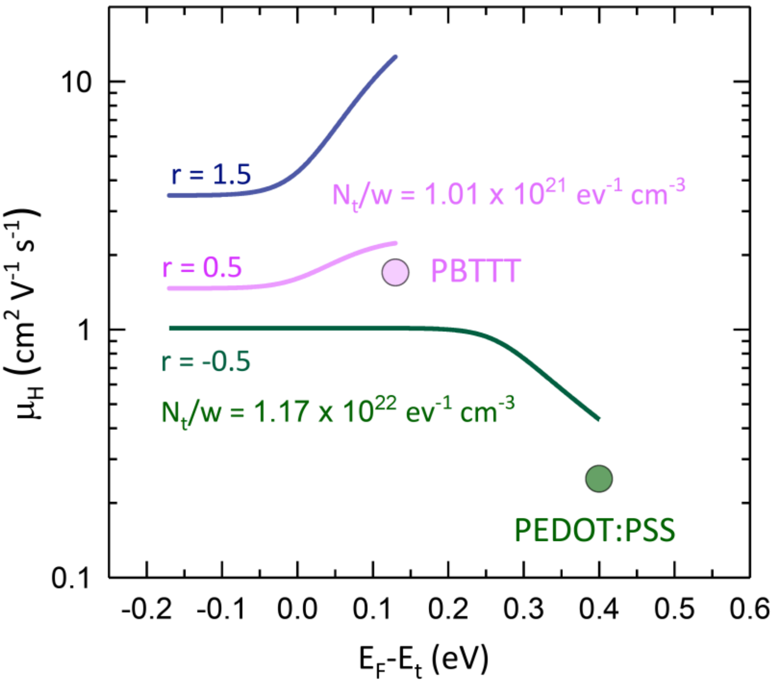

Supplementary Figure 12. Comparison of the calculated mobility with the experimental ones. Different classes of conducting polymers exhibit different scattering parameters, r

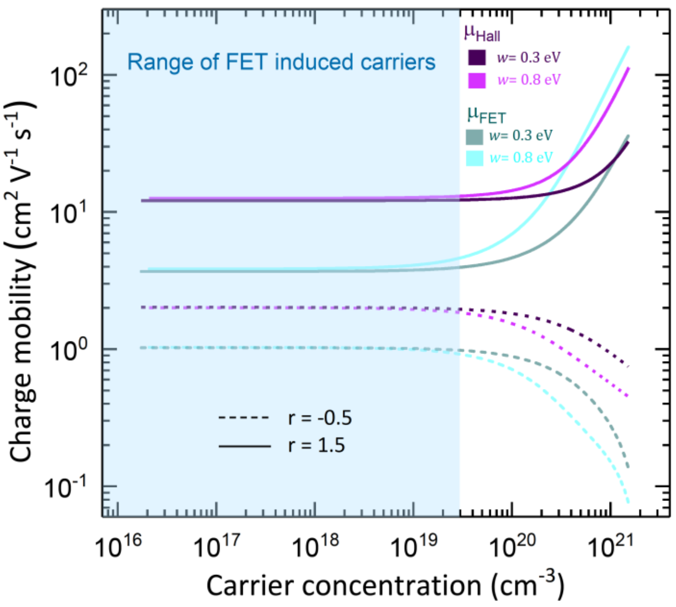

Supplementary Figure 13. The calculated Field-effect transistor (FET) and Hall mobilities. Mobilities are plotted as a function of carrier concentration at different values of w and r, and at fixed N_t.

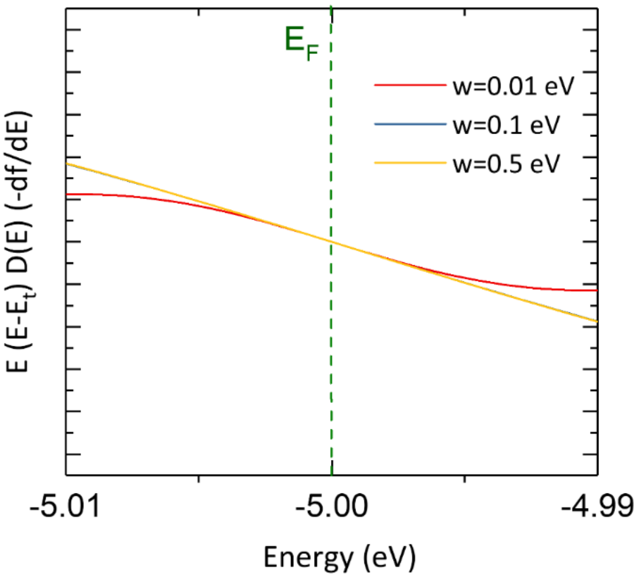

Supplementary Figure 14. Seebeck coefficient and DOS broadening. The function $E\ D(E)\left(-\frac{\partial f}{\partial E}\right)\ (E-E_t)$ that determines Seebeck coefficient (Equation 4 in the main text) as a function of energy. It can be noted that the integrand of this function with respect to energy has the same value at E_F regardless of w value, which explains why the Seebeck is not sensitive to w. Here, E_F is chosen arbitrarily to be at -5 eV.

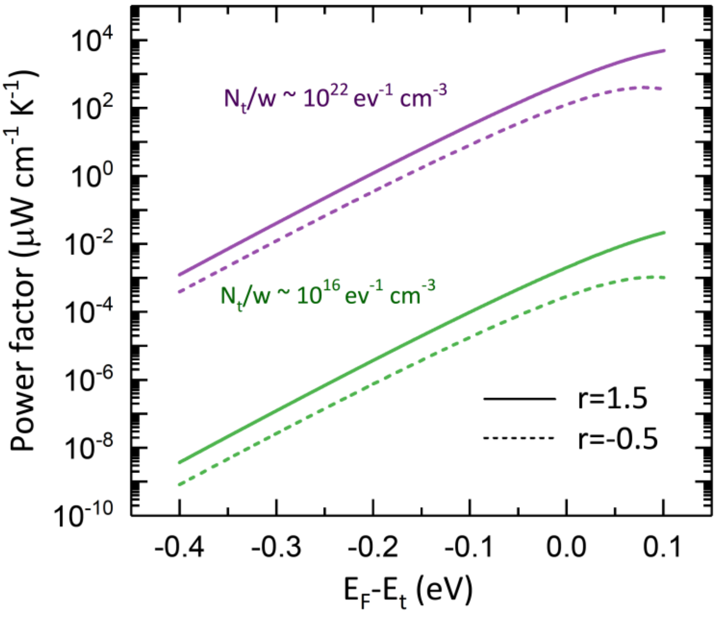

Supplementary Figure 15. Thermoelectric power factor ($S^2\sigma$). The power factor is calculated at different values of scattering parameters (r) and effective DOS (N_t/w).

Supplementary Tables

Supplementary Table 1. Hole deformation potential (DP) constants, E_1 and elastic constants, C_{ii} for P3HT along crystal axes a, b and c directions, respectively. The crystal axes, a, b and c are the directions of polymer chain, parallel chain and $\pi - \pi$ stacking, respectively.

	a	b	c
E_1 (eV)	10.8	0.446	2.45
C_{ii} (10^9 J m^{-3})	349	117	74.8

Supplementary Table 2. Comparison of experimental and theoretical Hall mobility at room temperature for two different polymers, PBTTT and PEDOT:PSS

Conducting polymer	σ_{exp} (S cm^{-1})	$n_{\exp(Hall)}$ (10^{20} cm^{-3})	$\mu_{\exp(Hall)}$ (cm^2 V^{-1} s^{-1})	$E_F - E_t$ (eV)	σ_{cal} (S cm^{-1})	μ_{cal} (cm^2 V^{-1} s^{-1})	n_{cal} (10^{20} cm^{-3})
PBTTT	170	5.3	1.7	0.13	172	2.2 ($r = 0.5$)	4.8
PEDOT:PSS	750	500	0.25	0.4	785	0.27 ($r = -0.5$)	180

Supplementary Note 1: Molecular Dynamics Simulations to Determine Paracrystallinity

Different configurations of P3HT were established to generate as wider range as possible of paracrystallinity:

Interdigitated configuration:

A 2×5×5 supercell was constructed and optimized without any cell constraint (Supplementary Figure 4). Using molecular dynamics (MD) simulations, the supercell was then annealed between 300–600 K for 200 heating cycles with 20 K heating and cooling steps (which means 15 heating steps to reach 600 K) and 0.5 ps of simulation at each temperature. 3 ns of total simulation time was applied.

Non-periodic configuration:

A non-periodic supercell was constructed by using 2×10×9 supercell (Supplementary Figure 5). The system was quenched with consecutive simulation and optimizations by using same method given for structure (i).

Fishbone-like configuration:

A different crystalline structure of P3HT with fishbone-like arrangement was used instead of highly packed comb-like arrangement (Supplementary Figures 4 and 5) as shown in Supplementary Figure 6. Lattice parameters are adopted from Colle *at al*[1]. A 2×5×5 supercell was optimized and annealed at same annealing parameters used in structure (i).

Mixed configurations:

Starting from five initial configurations containing comb-like and fishbone-like crystallites and then performing annealing on them, we can generate four new disordered configurations (Supplementary Figure 9). All cells have 60 chains, each chain has 10 monomers under periodic boundary conditions (ends of chains are bonded), each cell contains 600 monomers and exactly 15000 atoms. The annealing procedure is similar to the previous one.

It has been previously[2] shown that crystals possess g ~ $0-1\%$; semicrystalline systems, such as polymers, g is ~1−8%; while for highly disordered systems and melts, g exceeds 10%. The values of g for the generated systems range from 6.07$-$ 8.7%. Although we attempted various structural configurations of P3HT to generate a larger range of g, it is extremely challenging to generate structures with g> 10% using MD simulations.

Supplementary Note 2: Relaxation time calculations

All details related to the relaxation time calculations are mentioned the Methods in the main text. Supplementary table 1 below lists the relevant physical parameters for these calculations.

In order to confirm the validity of choosing τ_0 to be 10 fs, we have investigated some calculations as the following:

We find first the value of eta (η) from Supplementary Equation 10 ($n = \int D(E)\, f(E)\, dE$) that corresponds to carrier concentration of 10^{20} cm-3 (which is the same value used for relaxation time calculations). We find η = 6.26, at the following conditions (similar to ones used for P3HT fittings): w=0.4 eV; Et=-0.2 eV; r=1.5; N_t=6.4x10^{20} cm^{-3}. Then, by substituting η = 6.26 in Supplementary Equation 3 ($\tau(E) = \tau_0 \left(\frac{E-E_t}{k_BT}\right)^r$), we find τ= 157 fs, which is in the same range of τ values obtained from the relaxation time calculations (i.e. 203 and 106 fs). Therefore, τ_0 of 10 fs represents a reasonable number to use for fitting transport properties using BTE.

Supplementary Note 3: Transport properties:

Electrical conductivity

The electrical conductivity of charge carriers with energy (E) can be found from the generalized Boltzmann transport equation (Reference 15):

$$\sigma = \int \sigma_E \left(-\frac{\partial f}{\partial E}\right) dE \qquad \text{(Supplementary Equation 1)}$$

where f is the Fermi-Dirac distribution function; and σ_E is the transport function which has the following formula, under the energy-dependent relaxation time $\tau(E)$ approximation:

$$\sigma_E = \frac{2e^2}{3m^*} \tau(E) \,(E - E_t)\, D(E) \qquad \text{(Supplementary Equation 2)}$$

where e is the electronic charge; m^* is the effective mass of the charge carriers; $D(E)$ the electronic density of states (DOS); E_t is the transport energy below which charge carriers do not contribute to transport effectively, and it is given by $E_t = -0.49\, w$ (Reference 5), where w is the width of $D(E)$. The relaxation time, that is the average time between two scattering events, is determined by the dominant scattering mechanism through the scattering parameter, r:

$$\tau(E) = \tau_0 \left(\frac{E-E_t}{k_B T}\right)^r \qquad \text{(Supplementary Equation 3)}$$

and the prefactor, τ_0 is also ruled by the dominant scattering mechanism, and it can be found by solving the deformation potentials related to the scattering mechanisms (Reference 15). $D(E)$ is generally described in crystalline semiconductors as the following:

$$D(E) = \left(\frac{E}{k_B T}\right)^m \qquad \text{(Supplementary Equation 4)}$$

where m is the dimensionality factor and it has the values: -0.5, 0,and, 0.5 for 1D, 2D, and 3D systems, respectively. In the case of 3D DOS, and from Supplementary Equations 1, 2, and 3,the energy dependence of electrical conductivity will have this proportionality:

$$\sigma \propto E^{r+1.5} \qquad \text{(Supplementary Equation 5)}$$

the exponent, $r + 1.5$, is equivalent to the transport parameter (s) that was defined by Kang and Snyder (Reference 6). It is worth mentioning that, in our work, we distinguish between r and s as we apply the Gaussian DOS in our calculations, which does not require any dimensionality factor, hence r is more appropriate and accurate to use for describing transport properties.

Note, in the Kang-Snyder framework (Reference 6), the energy dependence of the transport function, $\sigma_E = \sigma_{E_0} \left(\frac{E-E_T}{k_B T}\right)^s$ depends on the transport parameter, s, which has the scattering parameter (r) embedded within, but since the exact nature of the DOS was not considered in their study, it was difficult to isolate the energy dependence of the scattering time (see Equation S25 in Reference 6).

Charge Mobility

Hall Mobility (μ_H)

Usually, charge mobility in semiconductors is found by using the following equation:

$$\mu_H = \sigma / ne \,, \qquad \text{(Supplementary Equation 6)}$$

which is valid only for metals and degenerate semiconductors. The general form of mobility is determined by the Hall coefficient (R_H) (which converges to $1/ne$ in the degenerate limit)[3]:

$$\mu_H = \sigma R_H \qquad \text{(Supplementary Equation 7)}$$

and:

$$R_H = \frac{3}{2e} \frac{\int \tau^2(E)\, E\, D(E) \left(-\frac{\partial f}{\partial E}\right) dE}{\left(\int \tau(E)\, E\, D(E) \left(-\frac{\partial f}{\partial E}\right) dE\right)^2} \qquad \text{(Supplementary Equation 8)}$$

Note that Supplementary Equation 6 does not account for energy-dependent scattering ($r = 0$). Supplementary Figure 11 shows R_H as a function of doping level for different values of r. It can be noted that R_H converges only in the highly degenerate regime as shown in the inset (yellow, red and blue colors). This means that in other regimes, one should consider the energy dependent scattering to find the exact value of μ_H.

By extending our analysis of Seebeck coefficient and electrical conductivity, where we show different polymers possess different r values, we can calculate the mobility by considering its energy dependence (Supplementary Equations 3, 7 and 8). For example, Kang *et al* (Reference 20) measured the room-temperature μ_H in PBTTT and PEDOT:PSS (1.7 and 0.25 $cm^2\ V^{-1}\ s^{-1}$, respectively) using the degenerate formula of mobility (Supplementary Equation 6). Using Supplementary Equation 1, one can find $E_F - E_t$ from the experimental conductivity values. The calculated mobilities using our model are consistent with the measured ones for PBTTT and PEDOT:PSS (see Supplementary Table 2).

Supplementary Figure 12 shows the calculated μ_H as a function of $E_F - E_t$ for different values of r, along with the measured μ_H of PBTTT and PEDOT:PSS as reported by Kang et al (Reference 20). PEDOT:PSS exhibits $r = -0.5$ which is consistent with our Seebeck-conductivity analysis (see Fig. 4 in the main text), while PBTTT shows $r = 0.5$ unlike what we found for other PBTTT and P3HT polymers that show $r = 1.5$ (see Fig. 4b). One possible explanation for this deviation in r, is that PBTTT is highly doped ($n \sim 5 \times 10^{20}$ cm^{-3} at room temperature), and hence, due to the high concentration of charge carriers in the backbones, some of them can be partially screened from the ionized anions residing in the side chains. Therefore, ionized impurity scattering is not the only scattering mechanism in charge transport, instead both ionized impurities and phonon scattering are working in parallel, resulting in $r < 1.5$.

In organic semiconductors, field-effect transistor mobility (μ_{FET}) is more attainable than μ_H. So, it would be more worthy to discuss the relationship of μ_{FET} with r and N_t/w. Supplementary Figure 13 shows μ_H and μ_{FET} as a function of carrier concentration at different values of r and w. μ_{FET} is found using the equation [4]:

$$\mu_{FET} = \frac{1}{e} \frac{d\sigma}{dn} \qquad \text{(Supplementary Equation 9)}$$

and taking the Gaussian DOS, n is given by :

$$n = \int D(E)\, f(E)\, dE \qquad \text{(Supplementary Equation 10)}$$

It is noted that μ_{FET} does not depend on w while r has the predominant effect on determining it, and this is supporting our analysis in Fig. 4c.

Thermoelectric Power Factor

Besides thermal conductivity (which is beyond the scope of our study), the thermoelectric power factor (PF) evaluates how efficient a material can be for thermoelectric applications. The power factor is defined by:

$$PF = S^2\sigma \qquad \text{(Supplementary Equation 11)}$$

where S is the Seebeck coefficient and σ is the electrical conductivity. Obviously, PF is more dependent on S which is directly proportional to (Supplementary Figure 15). Indeed, it is found, for the case of having effective DOS of N_t/w ~ 10^{23} eV^{-1} cm^{-3} (same case as in Fig. 4a in the main text), highest PF may be obtained at $r = 1.5$. In case, of having N_t/w ~ 10^{17} eV^{-1} cm^{-3}, such as in polymers with insulating side chains that do not count for total number of states (N_t), PF is orders of magnitude lower than the one for polymers with higher N_t. So, one promising route for obtaining highly thermoelectric-efficient polymers is to design polymers with unique molecular structure that leads to higher r and N_t/w.

Supplementary Note 4: Fitting parameters used in Seebeck-conductivity plots

Below are the fitting parameters used in the numerically solved Seebeck-conductivity plots in Figs. 4a and 4b in the main text using MATLAB:

```
Physical constants:
e=1.602e-19;         %electron charge (Coulomb)
tao0=1e-14;          %scattering time constant(s)
a=  1;               %relative effective mass
meff=a*(9.109e-31);  %effective mass (Kg)
kB=8.6173303e-5;     %Boltzmann constant (eV/K)
T=300;               %Absolute temperature (K)
E0=0;                %Peak position of Gaussian DOS
%PEDOT:Tos
r=-0.5
Ef0=linspace(-0.44,-0.02,1000)
Nt=2.08e21           %cm^-3
Et=-0.1 eV
w=0.2 eV
Eta = [-13.6, 3.2]
%PEDOT:Pss
r=-0.5
Ef0=linspace(-0.7,-0.02,1000)
Nt=1.52e21           %cm^-3
Et=-0.4 eV
w=0.8 eV
Eta = [-12, 15.2]
```

```
%P3HT and PBTTT upper limit
r=1.5
Ef0=linspace(-0.7,0,1000)
Et=-0.2 eV
Nt=6.4e18          %cm^-3
w=0.4
Eta = [-20, 8]
%P3HT and PBTTT lower limit
r=1.5
Ef0=linspace(-0.3,0,1000)
Et=-0.05 eV
Nt=2.96e16         %cm^-3
w=0.1
Eta = [-10, 2]
```

where eta is $\eta = (E_F - E_t)/K_B T$.

Supplementary Note 5: Chemical abbreviations

Fig. 4(a)

PEDOT:PSS → poly(3,4-ethylenedioxythiophene):polystyrene sulphonate
PEDOT:Tos → poly(3,4-ethylenedioxythiophene):tosylate

Fig. 4(b)

P3HT → poly(3-hexylthiophene)
PBTTT → poly(2,5-bis(3-tetradecylthiophen2-yl)thieno[3,2-b]thiophene)

Fig. 4(c)

P(NDI-T2) → poly(2,5-bis(3-tetradecylthiophen2-yl)thieno[3,2-b]thiophene)
PII2T[C, Si] → Poly{[N,N-9-bis(2-octyldodecyl)naphthalene-1,4,5,8-bis(dicarboximide)-2,6-diyl]-alt-5,59-(2,29-bithiophene)}, [C, Si]

DPP-BT [C, Se] → Diketopyrrolopyrrole-benzothiadiazole copolymer, [C, Se]

Supplementary References

1. Colle, R., Grosso, G., Ronzani, A. & Zicovich-Wilson, C. M. Structure and X-ray spectrum of crystalline poly(3-hexylthiophene) from DFT-van der Waals calculations. *Phys. status solidi* **248**, 1360–1368 (2011).
2. Yavuz, I., Martin, B. N., Park, J. & Houk, K. N. Theoretical Study of the Molecular Ordering, Paracrystallinity, And Charge Mobilities of Oligomers in Different Crystalline Phases. *J. Am. Chem. Soc.* **137**, 2856–2866 (2015).
3. Li, S. S. *Semiconductor Physical Electronics*. (Springer US, 2006).
4. Yi, H. T., Gartstein, Y. N. & Podzorov, V. Charge carrier coherence and Hall effect in organic semiconductors. *Sci. Rep.* **6**, 1–11 (2016).
